# Hydrogen Breath Test Dynamics Reflect Intestinal Fermentation Rather than Systemic Inflammation: A Data-Driven Diagnostic Analysis

**DOI:** 10.3390/diagnostics16132100

**Published:** 2026-07-04

**Authors:** Monika Waśkow, Magdalena Tańska, Sebastian Glowinski

**Affiliations:** 1 Institute of Health Sciences, Pomeranian University of Slupsk, 76-200 Slupsk, Poland; monika.waskow@upsl.edu.pl (M.W.); magdalena.tanska@upsl.edu.pl (M.T.); 2 Institute of Physical Culture, The State Academy of Applied Sciences in Koszalin, 75-582 Koszalin, Poland

**Keywords:** SIBO, hydrogen breath test, intestinal fermentation, gut microbiota, systemic biomarkers, inflammatory markers

## Abstract

**Background**: Hydrogen breath testing is commonly used to assess intestinal fermentation and diagnose small intestinal bacterial overgrowth (SIBO). However, it remains unclear whether hydrogen production reflects systemic inflammatory or metabolic status. This study evaluated the relationship between hydrogen production dynamics and systemic biomarkers using a data-driven analytical approach. **Methods**: This cross-sectional study included 162 adults undergoing lactulose hydrogen breath testing. Hydrogen production was characterized using continuous measures, including area under the curve (AUC), early and late hydrogen responses, and unsupervised clustering-derived hydrogen response groups. Associations with serum 25-hydroxyvitamin D, C-reactive protein (CRP), leukocyte count, and interleukin-6 (IL-6) were assessed using multivariable regression models adjusted for age and body mass index (BMI). **Results**: Hydrogen production showed substantial interindividual variability. Unsupervised analysis identified low-, intermediate-, and high-hydrogen response groups. Differences between groups were driven mainly by overall fermentation intensity rather than distinct temporal response profiles. No significant associations were observed between hydrogen production metrics and systemic biomarkers. Hydrogen-related variables were not independently associated with vitamin D, CRP, leukocyte count, or IL-6 concentrations. In contrast, BMI was consistently associated with inflammatory markers, particularly CRP and IL-6. Correlation analyses demonstrated strong relationships among hydrogen-derived variables but weak associations with systemic parameters. **Conclusions**: Data-driven analysis revealed marked heterogeneity in intestinal hydrogen production but no detectable association with systemic inflammatory or metabolic markers within the present cohort. These findings suggest that hydrogen breath test metrics primarily reflect local intestinal fermentation rather than systemic physiological status. Hydrogen breath testing remains useful for assessing gastrointestinal function, but no evidence supporting its value as a marker of systemic inflammation was identified in the present cohort.

## 1. Introduction

Small intestinal bacterial overgrowth (SIBO) is increasingly recognized as a disorder associated with abnormal colonization of the small intestine by bacteria that are typically found in the colon [1,2,3]. Excessive bacterial growth in the small intestine leads to intensified carbohydrate fermentation, resulting in increased production of intestinal gases, primarily hydrogen and methane [4,5,6]. In clinical practice, patients with SIBO commonly report a wide range of gastrointestinal symptoms, including bloating, abdominal pain, diarrhea, constipation, and excessive gas production [7,8]. Despite the growing interest in this condition, many aspects of SIBO pathophysiology and its potential systemic consequences remain poorly understood [9,10].

Hydrogen breath tests are currently among the most widely used non-invasive methods for assessing intestinal fermentation and diagnosing SIBO [10,11,12]. In particular, the lactulose hydrogen breath test is commonly applied, in which changes in hydrogen concentration in exhaled air are measured at specific time points following lactulose administration [13,14]. Interpretation of the results is typically based on simple threshold criteria, such as an increase in hydrogen concentration above a predefined value within a specified time after substrate ingestion [15]. However, this approach represents a considerable simplification of the complex fermentation processes occurring within the gastrointestinal tract.

Hydrogen production during breath testing is influenced by multiple physiological and microbiological factors, including intestinal transit time, gastrointestinal motility, availability of fermentable substrates, and the composition of the gut microbiota [16,17]. As a result, individuals classified within the same diagnostic category may exhibit markedly different hydrogen production profiles throughout the course of the test. Increasing evidence suggests that analyzing the dynamics of hydrogen production may provide additional insights beyond the conventional binary interpretation of breath test results [18,19]. Parameters reflecting the overall intensity of fermentation, such as the area under the concentration–time curve (AUC), as well as differences between the early and late phases of gas production, may better capture interindividual variability in fermentation processes among patients [20].

At the same time, increasing attention has been paid to the potential relationship between gut dysbiosis and systemic processes [21]. Alterations in the gut microbiota have been linked to chronic inflammation, metabolic disturbances, immune dysfunction, and abnormalities in nutritional status [22,23]. Some studies have also suggested possible associations between SIBO and levels of inflammatory markers, vitamin D, and hematological parameters [16,24]. However, it remains unclear whether hydrogen production measured during breath testing truly reflects broader systemic processes or primarily represents a marker of local fermentation activity within the gastrointestinal tract.

To date, most studies have focused primarily on the analysis of single breath test parameters or on classifying patients according to predefined diagnostic criteria [6,14]. Much less attention has been given to data-driven approaches aimed at identifying natural patterns of hydrogen production and assessing the continuous spectrum of fermentation variability among individuals [25,26]. Such an approach may allow for a more comprehensive characterization of fermentation processes and contribute to a better understanding of the potential relationships between hydrogen production and systemic parameters [27].

The aim of the present study was therefore to provide a multidimensional, data-driven characterization of hydrogen production dynamics during the lactulose breath test using unsupervised analytical methods and continuous parameters describing intestinal fermentation. In addition, relationships between hydrogen production patterns and selected systemic markers were evaluated, including C-reactive protein (CRP), leukocyte count, interleukin-6 (IL-6), and serum 25-hydroxyvitamin D levels. We hypothesized that hydrogen production demonstrates substantial interindividual heterogeneity and that a more detailed characterization of hydrogen response groups may provide insights not captured by the conventional threshold-based interpretation of breath tests.

## 2. Materials and Methods

### 2.1. Study Design and Participants

This cross-sectional study was conducted at the Interdisciplinary Center for Research on Civilizational Diseases at the Pomeranian University in Słupsk, Poland. The study included adults presenting with persistent gastrointestinal symptoms (including abdominal bloating, abdominal discomfort, altered bowel habits, or clinical suspicion of SIBO or other functional gastrointestinal disorders) who were referred for lactulose hydrogen breath testing. Although the cohort has been reported previously in the context of conventional SIBO classification, the present study addresses a distinct research question focused on data-driven characterization of hydrogen production dynamics using unsupervised analytical methods. No analyses presented in the current manuscript have been published previously. The present work focuses exclusively on data-driven characterization of hydrogen response dynamics and their relationship with systemic biomarkers.

**Figure 1:**
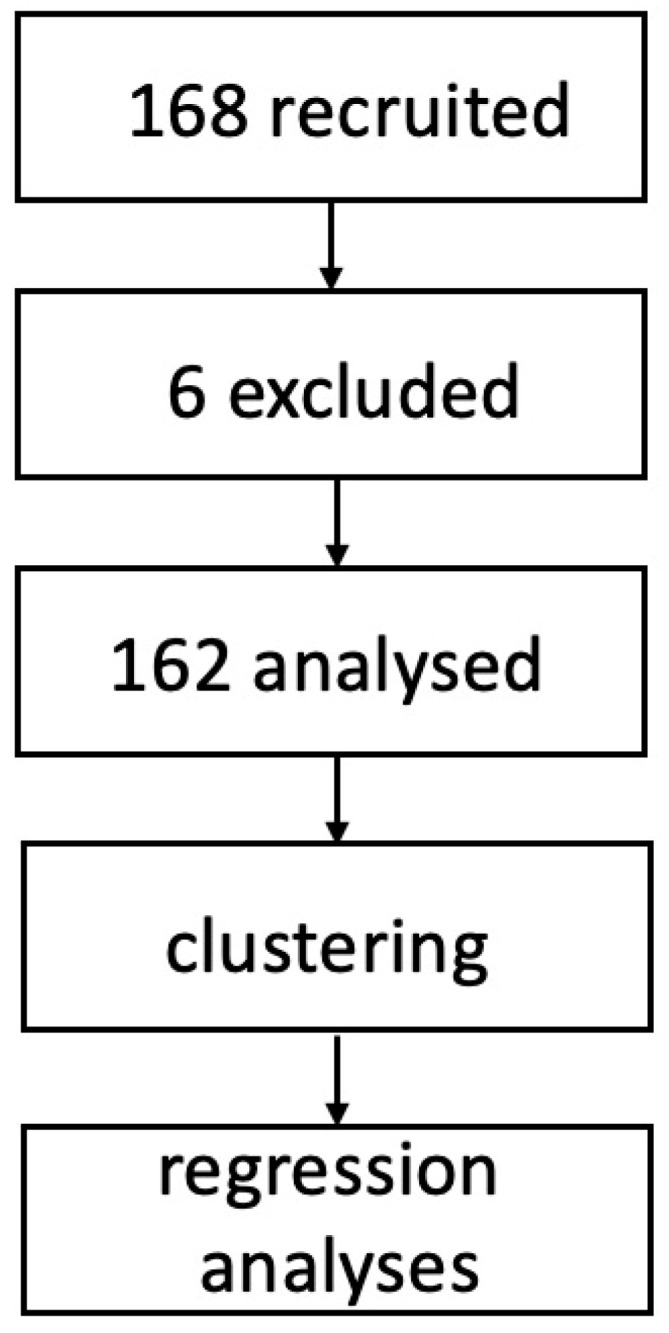
Flowchart of the experiment.

A total of 168 participants were initially recruited (Figure 1). After verification of eligibility criteria, 162 individuals were included in the final analysis. Inclusion criteria were age ≥ 18 years, the presence of gastrointestinal symptoms for at least three months, and written informed consent. Exclusion criteria included pregnancy, abdominal surgery within the previous six months, colonoscopy or fluoroscopy within four weeks before testing, antibiotic therapy within four weeks before examination, use of probiotics or prebiotics within two weeks before testing, smoking within 12 h prior to the examination, and non-compliance with pre-test preparation procedures.

All participants received standardized preparation instructions before breath testing. These included adherence to a restricted diet on the day preceding the examination and fasting for at least 14 h before the test. Smoking and chewing gum were prohibited for at least 12 h prior to testing.

The study was conducted in accordance with the Declaration of Helsinki. Written informed consent was obtained from all participants before enrollment. The study protocol was approved by the Bioethics Committee of the Medical Chambers in Gdańsk, Poland (Approval No. KB-15/23).

### 2.2. Measurements

Before hydrogen breath testing, participants completed a structured questionnaire to verify eligibility criteria and collect basic sociodemographic and clinical data. Anthropometric measurements were obtained using standardized procedures. Body weight and height were measured, and body mass index (BMI) was calculated as weight divided by height squared (kg/m^2^). Venous blood samples were collected after an overnight fast and analyzed in a laboratory accredited by the Polish Accreditation Centre using validated automated analytical platforms according to the manufacturers’ instructions. Hematological parameters were measured using the Sysmex XN analyzer (Sysmex Corporation, Kobe, Japan), routine biochemical analyses including CRP were performed using the Architect ci8200 (Abbott Laboratories, Abbott Park, IL, USA) and Cobas 8000 (Roche Diagnostics, Basel, Switzerland) systems, whereas serum 25-hydroxyvitamin D concentrations were determined using the Liaison XL chemiluminescence immunoassay analyzer (DiaSorin S.p.A., Saluggia, Italy). Participants followed the standard preparation protocol used in our center, including a 24-h dietary restriction, overnight fasting, and abstinence from smoking before the breath test.

All laboratory analyses were performed in an accredited clinical laboratory using standardized routine procedures in accordance with internal quality control standards and manufacturers’ protocols. The lactulose hydrogen breath test was performed using a Gastrolyzer Gastro+ device (Bedfont Scientific Ltd., Harrietsham, UK). Methane levels were not assessed. After collection of a baseline fasting breath sample, participants ingested 10 g of lactulose dissolved in 200 mL of water. Breath samples were then collected every 20 min over a 180 min period. Hydrogen concentrations were recorded in parts per million (ppm) at each time point. For analytical purposes, hydrogen production dynamics were characterized using parameters derived from the complete concentration–time profile, including total hydrogen production (AUC), peak hydrogen concentration, early hydrogen response (0–60 min), and late hydrogen response (80–180 min).

### 2.3. Statistical Analysis

All analyses were performed using R version 4.5.2 (31 October 2025) (R Foundation for Statistical Computing, Vienna, Austria) within the RStudio environment (version 2026.01.0+392, Posit Software, Boston, MA, USA). All available participants meeting the eligibility criteria were included in the analyses. Continuous variables are presented as mean ± standard deviation (SD) or median with interquartile range (IQR), as appropriate for data distribution. Prior to analysis, the dataset was screened for completeness, plausibility, and distributional characteristics. No missing data were present within the datasets used for the respective analyses. Normality of continuous variables was assessed using the Shapiro–Wilk test. Associations between hydrogen-related variables and systemic biomarkers were evaluated using multivariable linear regression models adjusted for age and body mass index (BMI). Multicollinearity was assessed using variance inflation factors (VIF). Model adequacy was evaluated through residual diagnostics, including assessment of linearity, homoscedasticity, and residual distribution.

To characterize patterns of hydrogen production, unsupervised hierarchical clustering was performed using three hydrogen-derived variables as input: total hydrogen production (AUC H_2_), early hydrogen response, and late hydrogen response. Prior to clustering, these variables were standardized (z-scores) to ensure equal contribution to the distance calculations. Euclidean distance was used as the similarity metric, and clusters were identified using Ward’s minimum variance linkage method. Cluster stability was assessed using the average silhouette width. Although a two-cluster solution showed the highest silhouette value, a three-cluster solution was retained for exploratory analyses because it provided a more informative representation of the continuous spectrum of hydrogen production. Principal component analysis (PCA) was additionally applied to visualize the structure of hydrogen production variability and facilitate interpretation of the dominant response patterns.

Correlation analyses were performed using Spearman correlation coefficients with two-sided significance testing. Because this study was exploratory and hypothesis-generating, adjustment for multiple comparisons was not applied. Consequently, the findings should be interpreted with appropriate caution and require confirmation in independent cohorts. Values of IL-6 below the limit of detection were handled using substitution with LOD/√2. Due to the skewed distribution of IL-6 concentrations, log-transformed values were used in regression analyses. All statistical tests were two-sided, and *p*-values < 0.05 were considered statistically significant.

## 3. Results

The final study sample included 162 adults referred for lactulose hydrogen breath testing because of persistent gastrointestinal symptoms, including abdominal bloating, abdominal discomfort, altered bowel habits, or clinical suspicion of small intestinal bacterial overgrowth (SIBO) or other functional gastrointestinal disorders. Participants covered a wide age range and were predominantly female. Baseline demographic characteristics according to conventional SIBO status are summarized in Table 1. Apart from age, no statistically significant differences were observed between the groups with respect to sex distribution (*p* = 0.68), body mass index (*p* = 0.84), waist circumference (*p* = 0.68), or CRP concentration (*p* = 0.57) (Table 1).

Exploratory clustering analysis identified three broad patterns of hydrogen production corresponding to low, intermediate, and high overall fermentation intensity. The low-, intermediate-, and high-hydrogen response groups comprised 69, 71, and 22 participants, respectively. The high-response group showed markedly elevated hydrogen production throughout the test, with a mean AUC of 12,771 ppm × min and increased values in both the early and late phases of the examination. In contrast, the low-response group was characterized by persistently low hydrogen concentrations across all time points (mean AUC: 1318 ppm × min). The remaining cluster demonstrated intermediate hydrogen production (mean AUC: 5620 ppm × min) with moderate early- and late-phase responses. Overall, the analysis revealed substantial interindividual variability in hydrogen production dynamics.

Cluster validity analysis indicated that a two-cluster solution provided the most stable partitioning of hydrogen response profiles, with an average silhouette width of approximately 0.52. Nevertheless, a three-cluster representation was retained for exploratory visualization because it improved interpretability of the continuous hydrogen production gradient observed across participants and was not intended to imply the existence of discrete biological response groups. Principal component analysis showed that the first principal component explained most of the variability in hydrogen dynamics and was driven primarily by overall hydrogen production intensity rather than by distinct temporal response patterns. The clustering analysis was exploratory and intended to identify broad patterns of hydrogen production rather than clearly separated response groups.

Although unsupervised analysis identified broad groupings of lower- and higher-hydrogen producers, distribution analyses revealed substantial overlap between individuals and a continuous right-skewed distribution of hydrogen production. Participants with higher hydrogen production exhibited consistently elevated fermentation activity throughout the test, particularly during the late fermentation phase, whereas lower producers showed attenuated but broadly similar temporal response patterns. Overall, the findings indicate that intestinal fermentation dynamics are better described as a continuous spectrum of fermentation intensity rather than as clearly separated biological response groups.

**Figure 2:**
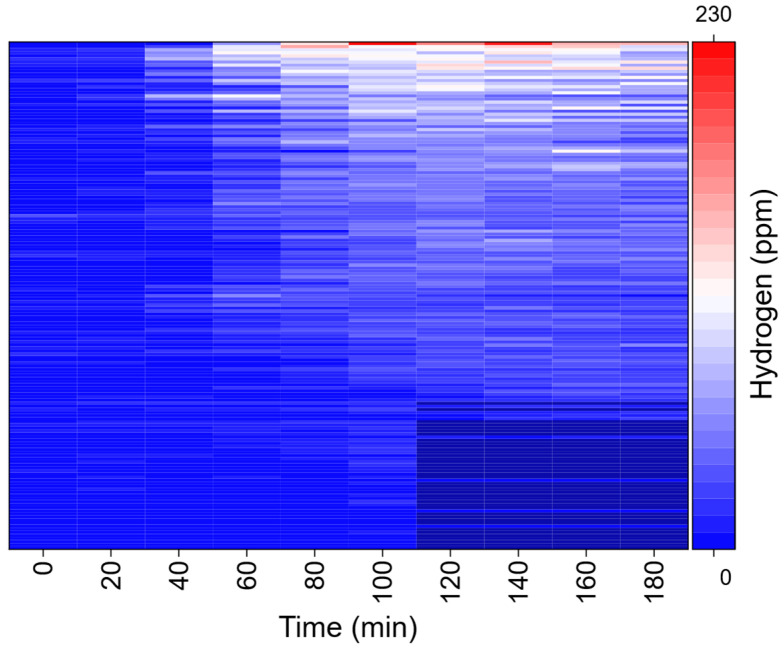
Heatmap of hydrogen production during the lactulose breath test. Participants are ordered according to total hydrogen production (AUC H_2_).

To further illustrate the continuous nature of hydrogen production variability, heatmap analysis of individual hydrogen response trajectories was performed. Participants were ordered according to total hydrogen production (AUC H_2_), revealing a gradual increase in fermentation intensity without distinct boundaries between response profiles. The visualization also showed that higher hydrogen production was mainly associated with greater late-phase fermentation activity rather than with fundamentally different temporal response patterns (Figure 2).

**Figure 3:**
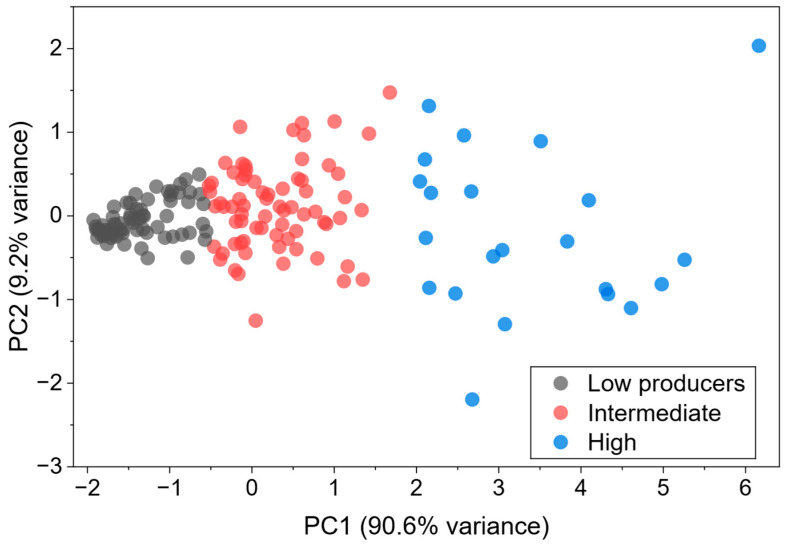
Principal component analysis illustrating the distribution of participants across exploratory hydrogen response groups. Abbreviations: PC1—first principal component; PC2—second principal component.

Principal component analysis showed that participants were distributed along a broad gradient of hydrogen production intensity rather than forming clearly distinct groups (Figure 3).

**Figure 4:**
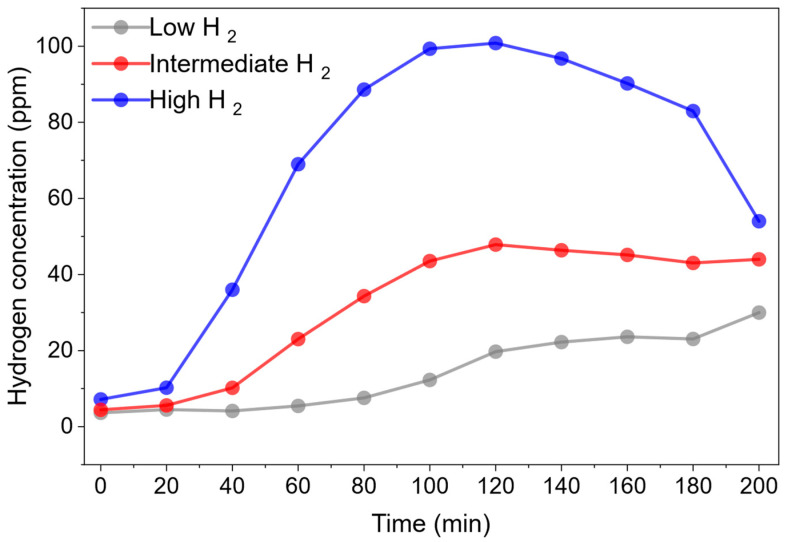
Mean hydrogen response curves for the exploratory hydrogen response groups.

Mean hydrogen (H_2_) response curves were plotted across exploratory hydrogen response groupings. Participants with higher hydrogen production showed consistently elevated hydrogen concentrations throughout the test, particularly during the late fermentation phase, whereas lower producers demonstrated attenuated but broadly similar temporal response trajectories (Figure 4).

Repeated-measures analysis demonstrated significant effects of both time (*p* < 0.001) and hydrogen response grouping (*p* = 0.0049) on hydrogen concentrations during the lactulose breath test. No significant group × time interaction was observed (*p* = 0.93). Differences between groups were therefore primarily attributable to overall hydrogen concentrations rather than differences in temporal response profiles.

**Figure 5:**
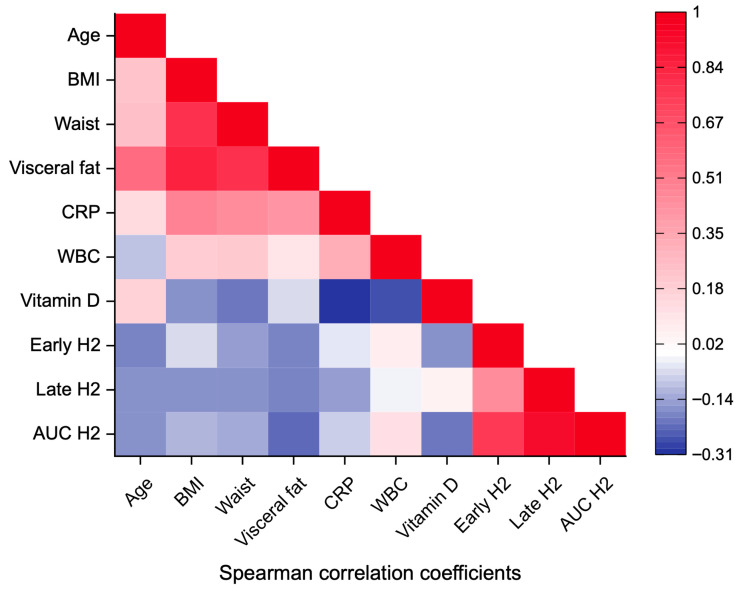
Spearman correlation heatmap demonstrating strong intercorrelations among hydrogen production metrics and weak associations between hydrogen production and systemic biomarkers. Abbreviations: BMI, body mass index; CRP, C—reactive protein; WBC, leukocyte count; AUC H2, total hydrogen production.

To further examine the relationships between hydrogen production dynamics, anthropometric measures, and systemic biomarkers, Spearman correlation analysis was performed (Table 2). Moderate-to-strong correlations were observed among hydrogen-derived variables (r = 0.73–0.96), whereas correlations between hydrogen production measures and inflammatory or anthropometric parameters were weak (|r| = 0.01–0.16) (Figure 5). Notably, the strongest correlations were observed among hydrogen-derived variables, whereas systemic biomarkers showed only weak correlations with hydrogen production measures. In contrast, BMI and CRP demonstrated a moderate positive correlation (r = 0.43).

**Figure 6:**
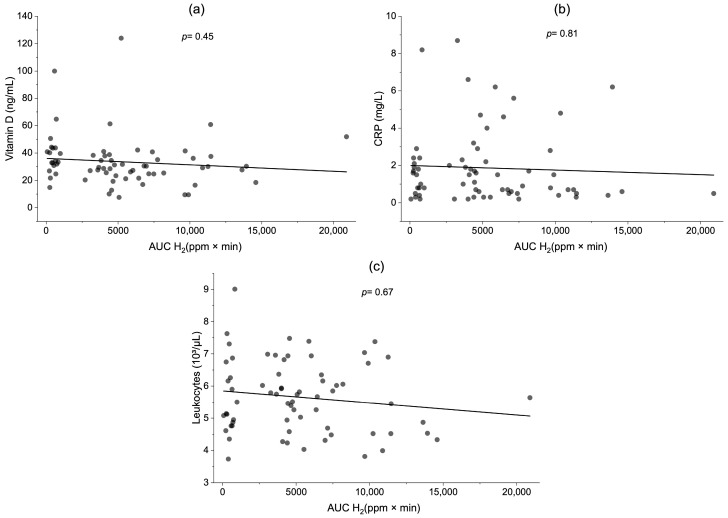
Association between hydrogen production (AUC H_2_) and systemic biomarkers: (**a**) vitamin D, (**b**) CRP (C-reactive protein), and (**c**) leukocyte count. No significant associations were observed (*p* = 0.45, 0.81, and 0.67, respectively).

Multivariable linear regression analyses adjusted for age and BMI showed no significant associations between hydrogen production (AUC H_2_) and systemic biomarkers. Specifically, AUC H_2_ was not associated with leukocyte count (β = 9.7 × 10^−6^, *p* = 0.666), vitamin D levels (β = −0.00039, *p* = 0.454), or CRP concentration (β = −1.02 × 10^−5^, *p* = 0.811). These null associations are illustrated in Figure 6, which shows scatterplots of AUC H2 against vitamin D, CRP, and leukocyte count, none of which demonstrated a significant relationship. For CRP, the estimated regression slope was −2.4 × 10^−5^ mg/L per ppm × min (95% CI: −1.37 × 10^−4^ to 8.8 × 10^−5^), indicating no evidence of a linear association between hydrogen production and CRP. In contrast, BMI was significantly associated with both leukocyte count (β = 0.062, *p* = 0.003) and CRP levels (β = 0.223, *p* < 0.001). BMI was significantly associated with leukocyte count and CRP, whereas hydrogen production (AUC H_2_) was not associated with any of the evaluated systemic biomarkers.

To further evaluate the relationship between intestinal hydrogen production and systemic inflammation at the cytokine level, multivariable linear regression analysis was performed with IL-6 as the outcome variable. After adjustment for age and body mass index (BMI), no significant association was observed between hydrogen production (AUC H_2_) and IL-6 concentrations (β = 9.34 × 10^−7^, *p* = 0.93). In contrast, BMI (β = 0.043, *p* < 0.001) and age (β = 0.008, *p* = 0.015) were independently associated with IL-6 levels. The overall model was statistically significant (R^2^ = 0.19, *p* < 0.001). BMI and age were independently associated with IL-6 concentrations, whereas AUC H_2_ was not. Similar results were obtained when hydrogen production was analyzed using peak hydrogen levels, confirming the absence of association with IL-6 concentrations (β = 6.7 × 10^−5^, *p* = 0.95).

Multivariable linear regression analyses adjusted for age and BMI demonstrated no significant association between exploratory hydrogen response groups and serum 25-hydroxyvitamin D concentrations. In contrast, BMI was inversely associated with vitamin D levels (β = −0.44), whereas age showed a modest positive association (β = 0.31). These findings suggest that host-related metabolic factors contribute more substantially to variability in vitamin D status than intestinal hydrogen production patterns.

Similar findings were observed for inflammatory markers. In multivariable regression analysis adjusted for age and BMI, hydrogen production response groups were not associated with CRP levels (β = 0.038, *p* = 0.94). In contrast, BMI showed a strong positive association with CRP concentration (β = 0.226, *p* < 0.001), indicating that systemic inflammation is primarily driven by host metabolic factors rather than intestinal fermentation patterns. Age was not significantly associated with CRP levels.

**Figure 7:**
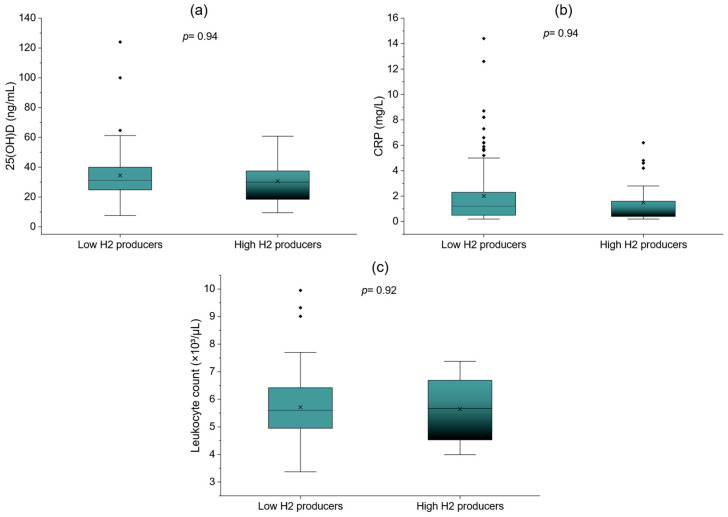
Comparison of systemic biomarkers between low- and high-hydrogen producers. (**a**) Serum 25-hydroxyvitamin D (25(OH)D concentrations), (**b**) C-reactive protein (CRP) concentrations, and (**c**) leukocyte count. Median (IQR) 25(OH)D concentrations were 31.1 (24.95–39.90) ng/mL in low H_2_ producers and 30.1 (18.50–37.50) ng/mL in high H_2_ producers. Median (IQR) CRP concentrations were 1.20 (0.50–2.30) mg/L and 0.60 (0.40–1.55) mg/L, respectively. Mean ± SD leukocyte counts were 5.73 ± 1.16 × 10^3^/µL and 5.69 ± 0.98 × 10^3^/µL, respectively. No statistically significant differences were observed between the groups. No statistically significant differences were observed between the groups (25(OH)D: *p* = 0.94; CRP: *p* = 0.94; leukocyte count: *p* = 0.92).

Consistent results were observed for leukocyte count. In multivariable regression analysis adjusted for age and BMI, hydrogen production response group was not associated with leukocyte levels (β = −0.025, *p* = 0.92). Hydrogen response group was not associated with leukocyte count, whereas BMI was positively associated with leukocyte count. Age showed a non-significant trend toward lower leukocyte levels. These findings are summarized in Figure 7, which illustrates the comparable distributions of vitamin D, CRP, and leukocyte count between the low- and high-hydrogen response groups.

Spearman correlation analysis demonstrated moderate-to-strong correlations among hydrogen production parameters. AUC showed very strong positive correlations with both early (r = 0.87, *p* < 0.001) and late hydrogen levels (r = 0.96, *p* < 0.001), indicating that these variables capture closely related aspects of hydrogen production dynamics. Early and late hydrogen values were moderately-to-strongly correlated (r = 0.73, *p* < 0.001), reflecting shared temporal features of fermentation. In contrast, no meaningful correlations were observed between hydrogen production metrics and systemic biomarkers.

Similarly, no significant association was observed between hydrogen production measures and IL-6 concentrations.

## 4. Discussion

The present study examined intestinal hydrogen production patterns using a data-driven analytical approach and evaluated their relationship with systemic biomarkers. Unsupervised clustering identified broad hydrogen response groups characterized mainly by differences in overall fermentation intensity. However, no detectable associations between these patterns and markers of systemic inflammation or metabolic status were identified in this cohort. The findings extend our previous observations from the same cohort and further indicate that hydrogen breath test metrics are only weakly related to systemic biochemical parameters.

In our previous study, SIBO defined according to conventional diagnostic criteria was not independently associated with vitamin D levels, inflammatory markers, or hematological parameters after adjustment for confounding variables [1]. The present study extends these findings by analyzing hydrogen production as a continuous process rather than relying solely on conventional diagnostic thresholds. Although unsupervised analyses identified broad patterns of hydrogen production, no detectable associations with systemic biomarkers were observed. Taken together, these findings suggest that hydrogen breath test responses primarily reflect local intestinal fermentation rather than systemic physiological processes.

One of the main findings of this study was the marked interindividual variability in hydrogen production dynamics. Exploratory clustering identified broad low-, intermediate-, and high-hydrogen response groups that differed mainly in overall fermentation intensity rather than in clearly distinct temporal response profiles. Previous studies have likewise emphasized the heterogeneous nature of SIBO and the substantial variability in both clinical presentation and microbial fermentation patterns among affected individuals [28].

These observations also underline an important limitation of conventional binary breath test classification systems, which may oversimplify the biological complexity of intestinal fermentation. Earlier studies have shown that hydrogen breath test results are influenced by multiple physiological and methodological factors, including intestinal transit, substrate availability, and microbial metabolism, potentially limiting the specificity of threshold-based diagnostic interpretations [29]. Nevertheless, even a more detailed characterization of hydrogen production patterns did not reveal meaningful differences in systemic biomarkers. This suggests that variability in hydrogen production is more closely related to local intestinal fermentation processes than to broader systemic physiological status.

Repeated-measures analysis indicated that differences between exploratory hydrogen response groups were predominantly quantitative rather than temporal. Participants with higher hydrogen production exhibited consistently elevated hydrogen concentrations throughout the test, but the overall shape of the response curves remained broadly similar across groups. This supports the view that intestinal hydrogen production reflects a continuous physiological spectrum driven mainly by differences in fermentation intensity rather than by distinct biological response patterns.

The absence of associations between hydrogen production and systemic biomarkers was consistent across multiple analytical approaches, including regression models, group comparisons, correlation analyses, and alternative measures of hydrogen dynamics such as peak hydrogen levels. Similar findings were observed for IL-6, indicating no detectable relationship between hydrogen production and cytokine-level inflammation. Although associations between SIBO and systemic inflammation have been reported in selected clinical populations, particularly in patients with cirrhosis [30], our findings did not support a similar relationship in this broader cohort of symptomatic individuals. This apparent discrepancy may be explained by differences in study populations. Many previous studies reporting associations between SIBO or gut dysbiosis and extra-intestinal manifestations, including metabolic syndrome, rosacea, or fibromyalgia, were conducted in patients with established systemic diseases or chronic inflammatory conditions. In contrast, the present study included symptomatic individuals referred for lactulose hydrogen breath testing, most of whom did not have severe systemic disorders. Furthermore, hydrogen breath testing reflects intestinal fermentation rather than the broader functional or compositional characteristics of the gut microbiota that may contribute to extra-intestinal manifestations.

Body mass index (BMI), in contrast, was consistently associated with inflammatory markers, whereas hydrogen-related variables were not. This suggests that systemic inflammatory status is influenced predominantly by host metabolic factors rather than by intestinal fermentation dynamics.

Notably, even participants representing the extremes of the hydrogen production spectrum showed remarkably similar levels of systemic biomarkers. Although hydrogen production differed substantially across response groups, CRP, leukocyte count, and vitamin D concentrations remained largely comparable. This observation further supports the interpretation that hydrogen breath test variability primarily reflects differences in intestinal fermentation intensity rather than systemic physiological status.

These findings also have practical implications for the interpretation of hydrogen breath tests in both clinical and research settings. Hydrogen breath testing remains a widely used non-invasive method for the assessment of intestinal fermentation and the diagnosis of SIBO. However, our results indicate that hydrogen-based measures—whether based on diagnostic thresholds, continuous variables, or data-driven response groups—have limited value as indicators of systemic metabolic or inflammatory status. This interpretation is consistent with current expert recommendations, which emphasize that breath testing should primarily be viewed as a functional measure of intestinal fermentation rather than a marker of systemic disease activity [31]. At the same time, these observations do not diminish the clinical utility of breath testing in the evaluation of gastrointestinal function, but they do argue against its broader interpretation as a marker of systemic effects.

From a diagnostic perspective, the present findings suggest that elevated hydrogen production during lactulose breath testing should not be interpreted as evidence of increased systemic inflammatory activity or adverse metabolic status. Although substantial variability in hydrogen responses was observed across participants, neither conventional hydrogen-related measures nor data-driven response groups were associated with CRP, IL-6, leukocyte count, or vitamin D concentrations. Clinicians should therefore interpret hydrogen breath test results primarily within the context of intestinal fermentation and gastrointestinal function rather than as surrogate indicators of systemic inflammation. These observations may help prevent overinterpretation of breath test findings and support a more focused application of hydrogen breath testing in the evaluation of SIBO and related gastrointestinal disorders.

Several factors may explain the lack of association between intestinal hydrogen production and systemic biomarkers observed in this study. First, hydrogen production reflects microbial fermentation of non-absorbable substrates within the intestinal lumen and therefore likely captures predominantly local gastrointestinal processes rather than host–microbiome interactions directly related to systemic physiology. Second, breath hydrogen levels are influenced by multiple physiological and methodological factors, including intestinal transit time, substrate availability, and microbial composition. Previous methodological studies have likewise shown that hydrogen breath measurements are strongly affected by transit dynamics and technical variability, which may limit their relationship with systemic physiological markers [14].

Another important limitation is the absence of methane measurements. Individuals with low hydrogen production may exhibit increased methane generation due to hydrogen consumption by methanogenic archaea. As a result, some participants may have been misclassified within lower hydrogen response categories, potentially obscuring underlying biological associations.

The complexity of the gut–systemic axis should also be considered. Although gut microbiota disturbances have been linked to systemic inflammation and metabolic regulation, these effects are likely mediated through mechanisms such as microbial metabolites, immune signaling pathways, and intestinal barrier function rather than through hydrogen production itself. Consequently, hydrogen breath testing likely does not capture the mechanisms linking intestinal microbiota with systemic physiology.

The strengths of this study include the use of a well-characterized clinical cohort and the application of several complementary analytical approaches. Unsupervised clustering enabled the identification of natural hydrogen production patterns without relying on predefined diagnostic thresholds, while regression and correlation analyses provided consistent support for the main findings. The study also extends our previous analyses performed in the same cohort, allowing for a broader evaluation of the relationship between intestinal fermentation and systemic biomarkers.

Several limitations should be acknowledged. First, the cross-sectional design precludes causal inference. Second, the study included only symptomatic individuals referred for lactulose hydrogen breath testing, which may limit the generalizability of the findings and introduce selection bias. Participants in the SIBO-negative group were older than those in the SIBO-positive group. Although age was included as a covariate in all multivariable regression models, residual confounding related to this baseline difference cannot be excluded. Women also predominated in the study population; therefore, caution is needed when extrapolating these findings to men. Although information on several clinical and lifestyle characteristics was collected, only age and BMI were included in the adjusted regression models, and residual confounding remains possible. Methane production was not assessed, precluding evaluation of intestinal methanogen overgrowth. Because methanogenic microorganisms consume hydrogen, some individuals with active intestinal fermentation may have exhibited relatively low hydrogen concentrations, potentially leading to physiological misclassification. The biomarker panel was limited to routinely available inflammatory and metabolic markers and did not include broader cytokine profiles, metabolic hormones, lipid-related indices, or fecal inflammatory markers. Therefore, subtle systemic associations may have remained undetected. Because this was an exploratory study, no adjustment for multiple comparisons was applied and the findings should be interpreted with appropriate caution. Hydrogen breath testing provides only an indirect measure of intestinal fermentation and does not directly assess microbial composition or activity. Consequently, the absence of detectable associations in this cohort should not be interpreted as evidence that such relationships do not exist in other populations or clinical settings. Finally, standardized symptom severity scores were not available. Therefore, the relationship between exploratory hydrogen response groups and symptom severity could not be evaluated. Future prospective studies incorporating validated symptom severity instruments are needed.

## 5. Conclusions

The present study did not identify detectable associations between intestinal hydrogen production patterns identified using data-driven approaches and systemic biochemical or inflammatory markers. Although substantial interindividual variability in hydrogen production was observed, no corresponding differences were detected in CRP, leukocyte count, IL-6, or vitamin D levels.

In contrast, systemic inflammatory markers were consistently associated with host-related factors, particularly body mass index, rather than with measures of intestinal fermentation. Within the present cohort, these findings suggest that hydrogen breath test responses primarily reflect local intestinal fermentation processes and have limited relevance as indicators of systemic inflammatory or metabolic status.

From a clinical perspective, the results support the role of hydrogen breath testing as a functional tool for assessing intestinal fermentation and SIBO-related physiology, while also indicating that its interpretation should remain focused on gastrointestinal processes rather than systemic effects.

Future studies combining breath testing with direct assessment of gut microbiota composition, microbial metabolites, intestinal permeability, and host immune pathways may help clarify the complex relationship between intestinal dysbiosis and systemic physiology.

## Figures and Tables

**Table 1:** Baseline demographic and clinical characteristics of participants according to SIBO status.

Variables	Negative (n = 62)	Positive (n = 100)	*p*
Age (SD) (years)	47.1 (13.3)	42.7 (11.8)	**0.0320** ^1^
Women, n = 131 (%)	49 (37.4)	82 (62.6)	0.6837 ^2^
Men, n = 31 (%)	13 (41.9)	18 (59.1)	
Body mass index (kg/m^2^)	25.4 (4.6)	25.1 (4.4)	0.6768 ^3^
Waist (cm)	86.1 (12.4)	85.7 (12.9)	0.8364 ^1^
CRP (mg/L)	1.95 (2.1)	1.92 (2.4)	0.5701 ^3^

Abbreviations: SD, standard deviation; CRP, C-reactive protein. ^1^ Student’s *t*-test; ^2^ Fisher’s exact test; ^3^ Mann–Whitney U test. Statistically significant values are shown in bold.

**Table 2:** Correlation matrix of hydrogen production metrics and systemic biomarkers.

	AUC	Early H_2_	Late H_2_	Vit D	CRP	WBC	BMI	AGE
AUC	1	0.87 ***	0.96 ***	−0.11	−0.08	0.03	−0.14	−0.12
Early H_2_		1	0.73 ***	−0.16	−0.07	0.05	−0.15	−0.11
Late H_2_			1	−0.06	−0.08	0.01	−0.14	−0.12
Vit D				1	−0.13	−0.18	−0.03	0.19
CRP					1	0.33 ***	0.43 ***	0.07
WBC						1	0.21 **	−0.09
BMI							1	0.21
AGE								1

Abbreviations: Values represent Spearman correlation coefficients. ** *p* < 0.01, *** *p* < 0.001. H_2_—hydrogen; Vit D—25-hydroxyvitamin D; CRP—C-reactive protein; WBC—leukocyte count; BMI—body mass index. Gray shading indicates statistically significant correlations.

## Data Availability

Available upon reasonable request.
